# Ivy Poisoning

**Published:** 1894-07

**Authors:** 


IVY POISONING will soon be seasonable ; and while it is well for people who resort to the country during the summer to be careful while they pick wild flowers, it may be of use to them to know that, according to a series of cases recently reported by J. Abbott Cantrell, M.D. (*Philadelphia Polyclinie,* May 12th), Labarraque's solution (solution of chloromated soda) is an excellent remedy. It should be applied in full strength by means of a pledget of lint or of diaper cloth, kept constantly wet with the liquid. It will effect a cure in from three to five days.—*The Sanitarian.*

